# Assessment of Respiratory Function and Need for Noninvasive Ventilation in a Cohort of Patients with Myotonic Dystrophy Type 1 Followed at One Single Expert Center

**DOI:** 10.1155/2022/2321909

**Published:** 2022-06-18

**Authors:** Carola R. Ferrari Aggradi, Elisa Falcier, Andrea Lizio, Alice Pirola, Jacopo Casiraghi, Alice Zanolini, Elena Carraro, Luca Mauro, Fabrizio Rao, Elisabetta Roma, Antonino Iannello, Elisa De Mattia, Andrea Barp, Sara Lupone, Valentina Gatti, Cristina Italiano, Valeria A. Sansone

**Affiliations:** ^1^The NEMO (NEuroMuscular Omniservice) Clinical Center, Milan, Italy; ^2^Neurorehabilitation Unit, University of Milan, Milan, Italy

## Abstract

**Introduction:**

Respiratory insufficiency is one of the main causes of death in myotonic dystrophy type 1 (DM1). Although there is general consensus that these patients have a restrictive ventilatory pattern, hypoventilation, chronic hypercapnia, and sleep disturbances, the prevalence of respiratory disease and indication for the effects of noninvasive ventilation (NIV) need to be further explored.

**Objectives:**

To describe respiratory function and need for NIV at baseline and over time in a cohort of adult patients with DM1.

**Methods:**

A total of 151 adult patients with DM1 were subjected to arterial blood gas analysis, sitting and supine forced vital capacity (FVC), peak cough expiratory flow (PCEF), nocturnal oximetry, and maximal inspiratory pressure and expiratory pressure (MIP/PEP).

**Results:**

On first assessment, 84 of 151 had normal respiratory function (median age: 38 years, median BMI: 23.9, and median disease duration: 11 years); 67 received an indication to use NIV (median age: 49 years, median BMI: 25,8, and median disease duration: 14 years). After a median time of 3.85 years, 43 patients were lost to follow-up; 9 of 84 required NIV; only 17 of 67 with the new NIV prescription were adherent.

**Conclusions:**

We provide additional data on the natural history of respiratory function decline and treatment adherence in a relatively large cohort of well-characterized patients with DM1. A high proportion (28%) was lost to follow-up. A minority (11%) required NIV, and only 25% were treatment adherent, irrespective of specific demographics and respiratory features. Our results also confirm previous findings, showing that age, disease duration, and higher BMIs are predisposing factors for respiratory impairment.

## 1. Introduction

Myotonic dystrophy type 1 (DM1) is a multisystem disorder characterized by multiple organ involvement [1]. Respiratory failure, together with cardiac involvement, is the main cause of death in adult-onset DM1 and is associated with increased morbidity and poor quality of life perception [2–4]. Respiratory function in DM1 patients is characterized by progressive inspiratory and expiratory muscle weakness, which causes a restrictive respiratory syndrome [4, 5].

Several authors have specifically addressed respiratory dysfunction in DM1 [6]. Most studies conclude that there is evidence that older age at onset and overweight are negative predictors of lung capacity and respiratory function [7, 8]. Less clear is the relationship to CTG size: in some patients, large expansions correlate with a decrease of vital capacity over time [9] or with expiratory muscle strength and oxygen saturation [10], while in some others, these values are reported as independent predictors of ventilator support [11]. Muscles of the trunk also play a role, and in particular, trunk flexor weakness has been associated with lower lung volumes [12].

While respiratory protocols for respiratory muscle tests are well defined [13, 14] as respiratory management protocols for patients with neuromuscular conditions in general [15, 16], the specific approach to respiratory treatment in DM1 is still challenging and management protocols for respiratory care in these patients vary [4]. This may be due to several reasons. Firstly, there are limited data on the natural history of changes in pulmonary function over time in patients with DM1 [4, 10]. Secondly, the clinical presentation of respiratory involvement varies between patients: in some patients, the muscle weakness may predominate, while in the majority, it is the CNS component, which may present as sleep-related breathing disorders including central apneas [17] and excessive daytime sleepiness that are independent of breathing issues and may not be alleviated by respiratory interventions such as CPAP, BPAP, or respiratory muscle training. Lastly, although there are several protocols recommending to use noninvasive ventilation (NIV) to correct for chronic respiratory insufficiency in DM1 [4, 18], the criteria to launch NIV in DM1 are variably applied, the effects of NIV on clinical symptoms vary [19–21], and overall patients' adherence is limited [4, 11, 19].

We present data on respiratory function in a large cohort of adult patients with DM1 followed at a single dedicated multidisciplinary neuromuscular center to analyze the prevalence of respiratory disease at baseline and over time and the indication for and effects of noninvasive ventilation (NIV).

## 2. Materials and Methods

### 2.1. Patient Population

**Figure 1:**
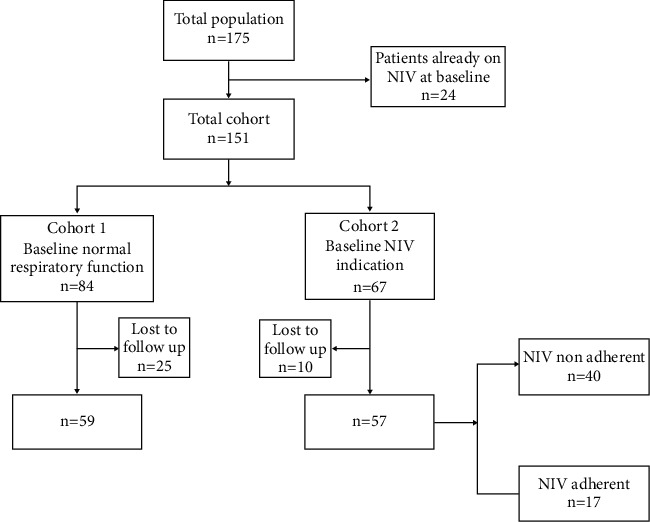
Respiratory follow-up of the 2 cohorts.

Demographical and medical records of 151 adult patients with genetically confirmed DM1 having at least one neurological and respiratory assessment were retrospectively reviewed from 2009 to 2019 at the NEMO Clinical Center in Milan, a specialized multidisciplinary clinic for patients with neuromuscular disorders and specifically with DM1. No a priori selection based on respiratory symptoms or signs was taken into account to enroll patients except that patients who were already on NIV (*n* = 24) were not included in the study cohort (Figure 1). Patients were seen as outpatients or inpatients at the NEMO Clinical Center. As outpatients, patients are seen every 6 months in a 1-day visit during which patients are subjected to neuromotor function tests, respiratory assessments, and psychological interviews. According to the patients' needs, additional nutritional and swallowing studies, cardiac monitoring, and orthosis assessments may be included. As inpatients, patients are usually admitted for 2- to 3-week neurorehabilitation programs, including respiratory assessments, as part of their care, and never during acute illnesses, of any kind.

Patients with a clear congenital (symptom onset within 1 month from birth) or pediatric onset (prior to 18 years of age) were excluded. Disease onset was taken as the age at which symptoms clearly consistent with DM1 (myotonia, muscle weakness, and cataracts) were documented in the charts. Patients with no signs or symptoms of myotonic dystrophy including no referred muscle weakness nor myotonia and a genetic diagnosis of DM1 were also included. It is common experience that weakness and myotonia may be present when examined and this is the reason for these inclusion criteria. Only patients with a clinically stable condition and especially no referral for acute pulmonary disease within the month prior to admission at the site were included.

### 2.2. Respiratory Assessments

All patients were subjected to a complete battery of respiratory assessments at baseline (*n* = 151) and after a median of 3.85 years (*n* = 116). All pulmonary function tests (PFTs) were carried out by the respiratory physiotherapists in compliance with the American Thoracic Society/European Respiratory Society guidelines [13, 22, 23], and the percentages of predicted values (%), age, weight, and height were recorded by the respiratory physiotherapist in occasion of every admission for follow-up. The PFTs were measured using a COSMED Quark PFT (Cosmed srl, Rome, Italy) spirometer and the decision whether to use the mouthpiece with nasal clip or the mask was based on the evaluation of each single patient, specifically on the eventual presence of facial muscle weakness and potential air leaks.

The PFTs included were the following tests:

- FVC in the seated and supine positions (FVC and sFVC, respectively) expressed both as absolute value, in liters, and as a percentage of the predicted value (FVC% and sFVC%, respectively) [22, 23].
- Peak cough expiratory flow (PCEF) expressed in L/min, and the maneuver was performed with a mask of fitting size, having the patient seated, and asking him/her to inhale as much air as possible and then to cough it out heavily. As per general and disease-specific guidelines, PCEF < 270 L/min was considered suggestive of a potentially ineffective cough, while a PCEF < 160 L/min signaled a high risk of pneumonia and respiratory failure [18].
- Maximal inspiratory pressure and expiratory pressure (MIP and MEP, respectively) expressed in cmH2O were performed using a mouth pressure meter MicroRPM (Micro Medical, Kent, UK) as a series of 3 to 5 maximal isometric respiratory maneuvers. MIP value was considered abnormal if <–60 cmH2O in absolute values, while MEP if <40 cmH2O [18].

All respiratory tests were then manually reviewed by a pulmonologist with expertise in neuromuscular disorders and especially in DM1.

Nocturnal cardiorespiratory monitoring was performed according to standard procedures [24], using the device Nox T3 (Nox Medical, Reykjavík Iceland, complete monitoring and eight channels), inclusive of a wrist saturimeter with a finger clip tip, either at patient's home the night before the visit (outpatients) or at the center itself (inpatients). The data considered relevant for clinical interpretation were the mean oxygen saturation (SpO2), the time spent with oxygen saturation <90% expressed as a percentage (T90), the oxygen desaturation index (ODI) during sleep, and the apnea-hypopnea index (AHI). The examination was considered abnormal either if the ODI was >15 events/h, the AHI exceeded 5/hour, and a sustained oxygen desaturation (SpO2) ≤ 88% for 5 consecutive minutes or SpO2 < 90% for >10% of total sleep time was registered [4, 18]. Thoracic and abdominal movements during sleep were also recorded. Arterial blood gas (ABG) analysis was performed at rest in the morning and collected by arterial puncture.

### 2.3. Criteria for NIV Indication

NIV was prescribed based on clinical and instrumental assessments according to ATS standards and the criteria, which were revised during the 207^th^ ENMC workshop [4]: AHI > 5/h, ODI > 15/h, FVC < 50% of predicted values, difference between FVC standing and lying (ΔFVC) ≥ 500 ml or 20% of the predicted value, pCO2 > 45 mmHg, and HCO_3_^−^ > 30 mmol/l. The criteria to prescribe NIV on first assessment were based on the combination of laboratory test results as per ATS guidelines [13, 18] and pulmonologist clinical assessments. Patients were defined as adhering to NIV if they used their ventilator for 4 hours or more per night and for at least 5 days a week, as downloaded from the device memory. Patients who were already on NIV on first assessment (*n* = 24) were excluded from the study.

### 2.4. Excessive Daytime Sleepiness Assessment

Excessive daytime sleepiness was self-reported using the Epworth Sleepiness Scale [25] (ESS). Sleepy patients were considered as such if scores were >10. This was available in 76 of 151 charts only.

### 2.5. Neuromotor Assessments

Muscle disease severity was rated using the Muscle Impairment Rating Scale (MIRS), an ordinal 5-point rating scale, able to track the clinically recognized distal to proximal progression of the muscular involvement in DM1, partly implementing the manual muscle testing of 11 muscle groups [26]. Additionally, the distance performed during the 6-minute walk test (6MWT) and the pre- and post-test dyspnea scales were collected for ambulatory patients.

### 2.6. Coping Strategy Assessment

The COPE-25 questionnaire [27] was administered to see whether coping strategies could at least in part come into play in determining adherence to management protocols in respiratory care.

### 2.7. Statistical Analysis

Shapiro–Wilk test and Levene's test were used to assess the normality of the distribution and the homogeneity of the variance. Data are summarized as median and interquartile ranges for continuous variables, and as numbers and percentages for categorical ones.

The nonparametric Mann–Whitney *U* test and the chi-squared/Fisher's exact test were used to compare patients without NIV indication and patients with NIV indication in terms of continuous and categorical demographic, clinical, and respiratory features, respectively.

The correlations between respiratory features and demographic and clinic characteristics were investigated using the Spearman's rank correlation coefficient.

To standardize the trend of decline between groups over time, only patients who attended at least 2 visits within 6 years were considered. The decline for each variable was calculated for each patient from the time of the first recording to the last recording, assuming a linear progression. The average annual decline for each variable was then calculated and compared between patients without NIV indication and patients with NIV indication at baseline using the nonparametric using the Mann–Whitney *U* test. The same analysis was also used to compare the average annual decline between NIV nonadherent and NIV adherent patients.

The Mann–Whitney *U* test was also used to compare the COPE-25 scores between patients without NIV indication and patients with NIV indication at baseline, and between NIV nonadherent and NIV adherent patients.

Finally, all the comparisons between patients with and without follow-up data were investigated using the chi-squared/Fisher's exact test when the IVs were categorical, and the Mann–Whitney *U* test when the IVs were continuous.

All tests were two-tailed, and a *p* value <0.05 was considered as statistically significant.

All the analyses were performed using SAS 9.3 (SAS Institute Inc.)

## 3. Results

### 3.1. Demographic and Clinical Features at Baseline

Demographic and clinical details are provided in Table 1. Both men and women were equally distributed (56% women) with BMI values ranging from normal to overweight ranges. The majority were mildly to moderately affected patients (MIRS 3 and 4), with CTG ranging from moderate to large expansions and with, at least, a 6-year history of disease. Only 4 patients were sitters, while 82 of 103 (79.6%) patients in whom the 6MWT was available in their charts were able to walk for more than 300 meters.

### 3.2. General Respiratory Features and NIV Indication at Baseline

Table 2 describes the general respiratory features at baseline of the entire cohort. Sitting FVC was below normal (≤80%) in about half of our patients (77 of 151) and 67 (44.4%) had an indication for NIV at baseline according to one or more of the aforementioned criteria. Demographic and general clinical features were compared between patients having normal respiratory baseline assessments (*n* = 84, cohort 1 in Table 3) and patients with indication to use NIV at night (*n* = 67, cohort 2 in Table 3). Results are summarized in Table 3. Significant older age, more severe muscular impairment, and longer disease duration characterized the patients who received an indication to start NIV. This was in line with data of patients who were already on NIV on first assessment at our site (data not shown and not considered in this analysis). Three of 84 patients (3.6%) with no indication for NIV at baseline, and 10 of 67 (14.9%) receiving an indication to use NIV had a PM or an ICD.  Table 4 specifically compares the respiratory features of patients with no indication for NIV at baseline and of those receiving a new NIV prescription. Details for each patient are provided in e-Table 1.

Obstructive sleep apneas (OSA) were recorded in a minority (4.6%). Excessive daytime sleepiness was reported by 24.2% of patients (*n* = 16 of 66 available ESS scores). Of these 16 patients, 9 patients reported daytime sleepiness, none reported morning headaches or snoring during the collection of medical history specifically addressing respiratory symptoms, 1 patient reported both dyspnea at rest and exertional dyspnea, and 2 patients reported subjective fatigue, with some symptoms overlapping in some patients.

Twenty-three of 151 patients (15.2%) fulfilled criteria for secretion management support (air-stacking or cough assist) based on peak cough expiratory flow (PCEF) ≤ 270 L/min, as suggested in the most recent consensus on respiratory management of DM1 patients [18].

Respiratory and demographic details for each patient are provided in e-Table 1.

### 3.3. Follow-Up of the Whole Cohort

The median follow-up period between first and last recording was 44.90 months [26.32–59.20 months, 3.74 years]. Thirty-five patients of 151 were lost to follow-up as indicated in Figure 1. These were patients mainly having a MIRS of 2. No other demographic features distinguished these patients lost to follow-up compared with the rest of the cohort. Considering respiratory and motor assessments, patients lost to follow-up reported a significantly higher ODI and had a significantly lower pH, FEV1/FVC ratio, PCEF, and MEP, and the distance they were able to walk during the 6MWT was significantly less compared with the rest of the cohort.

Eleven of the 116 patients (9.5%) available for follow-up lost their ability to walk. None developed cardiac symptoms or signs requiring pacemaker or implantable cardiac defibrillators of other cardiac pharmacological treatments during our observational period.

### 3.4. Respiratory Function over Time

Table 5 describes the changes per year of the nocturnal oximetry, morning ABG, and spirometry parameters from the whole cohort. No clinical meaningful changes were observed in general for all parameters.

Of the 116 patients available for follow-up (last observational visit), 59 still maintained a normal respiratory function at baseline and 57 had received an indication for NIV at night at baseline. The observational period is comparable between the study groups (38.10 months [24.13–55.60] for those with normal respiratory function at baseline and 46.17 months [31.17–60.83] for those receiving a prescription for NIV at night at baseline).

Nine of the 59 patients (15.3%) who had normal respiratory tests and pulmonary assessments at baseline fulfilled criteria for NIV indication at night on follow-up. No specific clinical and demographic features characterized these 9 patients, who were similar to the remaining patients maintaining normal respiratory function during the same observational period. NIV was prescribed in these patients due to abnormalities registered in those parameters that the latest guidelines and consensus [4, 19] recognized as indicators for a timely initiation of ventilator support at night in this population. In detail, 2 patients registered an AHI > 5/h; 1 had an ODI > 15/h; FVC < 50% of predicted was registered in 1 patient who also had Δ ≥ 20%; pCO2 > 45 mmHg in 3 subjects; and HCO3^−^ > 30 mmol/L in 8 patients. Additionally, being the overall respiratory status of each subject analyzed by a pulmonologist specialized in DM1, 2 patients whose respiratory assessments were considered “borderline” were initiated to NIV in a more proactive and preventive way. Respiratory details of these 9 patients are provided in e-Table 1.

### 3.5. NIV Effects and Adherence

Mean SpO2 and ODI on oximetry and pCO2 on ABG improved in the patients who started NIV based on symptoms and respiratory test results (cohort 2, Table 6). Regarding the min SpO2, pO2, and HCO3, no significant differences were observed among patients who had indication for NIV and those with normal respiratory function on first assessment. Only 17 of 57 with indication for NIV however used their ventilator regularly. Comparing the results between the 17 adherent and the 40 nonadherent patients in cohort 2 (fulfilling criteria for NIV at baseline), we observed that there was a statistically significant improvement in both min SpO2 and pO2 in the adherent cohort (Table 7). General clinical features including sex, age, disease duration, BMI, and MIRS scores were similar between the 2 groups. Details of the correlations between respiratory parameters and demographic and clinical features are provided in e-Table 2.

Follow-up data prior and after NIV use was available in only 7 patients of the 9 who had shifted from normal respiratory parameters to NIV at night. When we compared the first available evaluation of patients who shifted from normal respiratory parameters to NIV during the follow-up period with the respiratory parameters of the group of patients who reported a normal respiratory function during the entire follow-up period, the patients requiring NIV proved to have a significantly longer disease duration (18.00 [12.00–25.00] vs 10.00 [5.00–16.50], *p* = 0.0165) were more significantly impaired based on MIRS scores (100% of patients above 2 points compared with the 54.7% of the normal respiratory function group, *p* = 0.0088) and had a significant worse respiratory function in terms of mean SpO2 and pH (93.40 [62.50–95.70] vs 96.40 [94.80–97.30], *p* = 0.0235; and 7.44 [7.42–7.47] vs 7.48 [7.44–7.49], *p* = 0.0194, respectively). The 9 patients who shifted to NIV reported a feeling of well-being and reduced tiredness on clinical history, which was the reason why they were able to use their ventilators at night continuously for 4 hours or more when we discussed the results with these patients.

No difference was observed in the coping strategies assessed by the COPE-25 questionnaire (seeking social support for instrumental reasons, behavioral disengagement, positive reframing, active coping, and turning to religion) between NIV adherent and nonadherent patients.

## 4. Discussion

Our results provide additional data on respiratory parameters in a large cohort of well-characterized adult patients with DM1 and suggest that respiratory function, in general, may have a slow rate of progression. Only a minority of our patients developed the need for NIV during the observational time frame of the study. Our results also confirm that respiratory dysfunction is frequent in DM1, that there are distinctive demographic and clinical features in patients requiring NIV compared with those with normal respiratory function at baseline, that referral for typical respiratory symptoms like shortness of breath or orthopnea is unusual, and that adherence to NIV is a limiting factor.

There is general consensus that DM1 is a slowly progressive disorder [1–4], but the rate of decline of the multiple organs involved still needs to be defined. During our observational period, respiratory parameters overall did not show a clinically meaningful change in the cohort taken as a whole, in line with other reports [5, 6]. Only 9 of 84 (11%) patients who had normal respiratory parameters at baseline (cohort 1) developed the need for NIV at night during our observational period. We could not detect any distinctive clinical or respiratory features in these 9 patients that could help to predict a potential break point between normal and dysfunctional ventilation in patients with DM1. Unfortunately, the follow-up period and sample size did not allow to appreciate any significant improvement or delay in progression.

In line with previous reports describing the restrictive pattern of respiratory involvement and the predictive factors of respiratory involvement in this disease [6], we provide additional data on the prevalence of respiratory disease in DM1. The majority of the patients in our cohort were mildly to moderately affected, more than 60% could walk for more than 300 meters in 6 minutes, and only 11 were sitters. Yet, half of the patients when first assessed at baseline had an FVC below normal and had an indication for nocturnal noninvasive support. The patients with an indication for NIV were significantly older, had a longer disease duration, were more severely disabled (higher MIRS scores), and had higher BMI scores compared to the ones with normal respiratory function (cohort 1), supporting previous evidence [6]. Of note, that none of our patients, including those with FVC below normal, referred to our site for shortness of breath or orthopnea. Less than a quarter reported excessive daytime sleepiness on the Epworth Sleepiness Scale, but this was not a reason for referral, nor was choking in those patients with weak peak cough flow, which is usually associated with secretion management difficulties. This is not unexpected considering the passive attitudes and apathy of many patients with DM1 [28, 29], but it also emphasizes the complexity of respiratory dysfunction in this disorder, where central nervous system-related problems such as sleep apneas, sleep-related breathing disorders, and central fatigue and sleepiness play a predominant role [30, 31].

When we consider the changes over time in the patients who received an indication to start NIV at night at baseline (cohort 2), we can observe that, as expected, respiratory parameters improved. Patients reported a general feeling of well-being with no specific referral to respiratory symptoms who were never the cause for referral nor the main and only reason to start NIV. This is in contrast to the reports in 12 patients on and off NIV after 1 month [32] in whom no changes in respiratory parameters were observed and the patients experienced very little benefit in symptoms or quality of life. The observational period in the report of the 12 patients was however very short (1 month). In fact, Boussaid and coworkers [8] described an increase in the risk of major events (invasive ventilation and death) in a large cohort of patients who should have used NIV at night in a 10-year observational period. In our cohort of patients who regularly used NIV, pCO2 increased. We can speculate that this rise may be due to a more severely impaired central drive for hypercapnia and hypoventilation control, which progresses despite the strength of the respiratory pump, which is what NIV mainly acts on.

Despite improvement in respiratory parameters, adherence to NIV prescription was limited to a minority of patients in agreement with previous reports and clinical experience [4, 8]. We can assume that the limited compliance was not due to the type of ventilator used, the type of interface, or the leaks because several attempts were made by therapists and pulmonologists with long-lasting experience in neuromuscular disorders and in DM1, and an inpatient setting was also provided for the most difficult cases. We were unfortunately unable to detect specific distinctive features between adherent and nonadherent patients [4, 7, 33]. Considering previous studies describing major events in the long term for patients who should have used NIV but were not adherent to this prescription [8], we recommend caution in amending the current recommendations to initiate NIV in these patients. Further studies are needed to determine whether additional strategies (home care to monitor NIV use where specialized nurses or PTs could also play an educational role on improving patients sleep-wake cycle, refresh training, and provide more explicit and accessible information on the effects of NIV adherence) could actually improve adherence in this patient population.

No quality-of-life data and disease burden perception were captured in this study, and this represents a major limitation to the interpretation of compliance to NIV and to respiratory dysfunction perception. Although we included the COPE questionnaire in the study, no detailed neuropsychological testing was performed to define the cognitive and behavioral profile of our study population, which could provide information on potential additional management strategies we could have adopted. Moreover, the retrospective nature of the study did not allow to include sleep studies to identify those patients with predominant sleep-related breathing disorders in whom the CNS component predominates and NIV has minimal effects on the central fatigue and sleepiness. Another limitation due to the retrospective nature of the study was the variable availability of the data and missing information between baseline and final assessments led to the need to consider progression as linear, but this may not be the case for all patients [5]. Our observational period was also probably not long enough to capture long-term major events of patients who should have used NIV but were not adherent to this prescription. Considering previous studies describing major events in the long-term for patients who should have used NIV but were not adherent to this prescription [8], we recommend caution in amending the current recommendations to initiate NIV in these patients. Further studies are needed to determine whether additional strategies could actually improve adherence in this patient population (home care to monitor NIV use where specialized nurses or PTs could also play an educational role in improving patients' sleep-wake cycle and refresh training and provide more explicit and accessible information on the effects of NIV adherence).

In conclusion, we present data on respiratory function over a relatively long period of time in a large cohort of well-characterized adult patients with DM1 and confirm that respiratory dysfunction is frequent and complex. While we confirm that the older and more disabled patients with a longer disease duration are the ones usually requiring NIV, we provide evidence that patients may develop the need for nocturnal NIV support no matter the lack of respiratory symptoms and their degree of muscular disability. Although a minority is adherent to respiratory treatment and hypercapnia persists, the correction of the oximetry profile following NIV introduction and use may, at least in part, contribute to the general improvement of well-being described in the patients available in this study. In line with other reports [29, 33], despite the limitations with adherence and the complexity to assess benefit given the role of the central component, we emphasize the need to recommend respiratory tests on first assessment and to closely monitor nocturnal oximetry and hypercapnia in the older, weaker, and with higher BMI patients, despite their symptoms.

## Figures and Tables

**Table 1:** Demographic and clinical features of the whole cohort (*n* = 151).

Age	43.00 [34.00–52.00]

Sex, *n* (%)	
Male	67 (44.4)
Female	84 (55.6)

MIRS, *n* (%)	
1	9 (6)
2	36 (23.8)
3	50 (33.1)
4	52 (34.4)
5	4 (2.7)

Disease duration	13.00 [6.00–19.00]
BMI	24.82 [21.71–27.51]
CTG	600 [400–920]
Sitters, *n* (%)	4 (2.7)
PM/ICD, *n* (%)	16 (10.6)

All data are presented as median and interquartile ranges, except where otherwise indicated. Patients were classified as “sitters” if unable to walk for 10 meters without support. MIRS, Muscle Impairment Rating Scale; BMI, Body Mass Index; PM, pacemaker; ICD, implantable cardiac defibrillator.

**Table 2:** Respiratory features of the whole cohort at baseline (*n* = 151).

	*n*	Median [IQR]
Nocturnal oximetry		
Mean SpO2	128	94.35 [92.35–96.30]
Min SpO2	122	84.00 [77.00–89.00]
ODI	121	4.30 [1.30–15.20]

Morning ABG		
pH	121	7.45 [7.43–7.48]
pCO2	122	42.20 [38.30–46.60]
pO2	122	81.85 [70.40–92.60]
HCO3-	121	29.90 [27.80–32.70]

Spirometry		
FVC % sitting	151	80.00 [67.00–97.00]
FVC % supine	131	79.00 [63.50–89.00]
FEV1%	129	76.00 [63.00–91.10]
FEV1/FVC	129	0.80 [0.76–0.84]
∆ FVC %	131	6.00 [2.00–10.00]
PCEF	149	323.00 [272.40–402.00]
MIP	58	56.50 [37.00–79.00]
MEP	58	69.00 [50.00–102.00]

6MWT		
Meters	103	391.00 [315.00–500.00]
Mean O2 saturation	74	95.00 [93.00–97.00]
Pretest dyspnea	78	0.00 [0.00–0.00]
Post-test dyspnea	78	2.00 [1.00–4.00]

NIV indication		
No indication	84	55.6^*∗*^
Baseline indication	67	44.4^*∗*^

Secretion management		
Air stacking, yes	15	9.9^*∗*^
Cough assist, yes	8	5.3^*∗*^

IQR, interquartile range; SpO2, oxygen saturation; ODI, Oxygen Desaturation Index; pH, potential of hydrogen; pCO2, partial pressure of carbon dioxide; pO2, partial pressure of oxygen; HCO3-, bicarbonate; FVC, forced vital capacity; FEV1, forced expiratory volume in 1 second; PCEF, peak cough expiratory flow; MIP, maximal inspiratory pressure; MEP, maximal expiratory pressure; 6MWT, six-minute walk test; NIV, noninvasive ventilation. Pretest dyspnea and post-test dyspnea were calculated using the modified dyspnea Borg scale. This scale asks the patient to rate the difficulty of breathing from 0 (no difficulty) to 10 (maximal difficulty) at the very beginning of the 6MWT and immediately after. ^*∗*^percentage.

**Table 3:** Clinical and demographic comparison between cohorts according to NIV indication.

	Cohort 1 (*n* = 84)	Cohort 2 (*n* = 67)	*p* value
	No NIV indication	NIV indication at baseline	
Age	38.00 [29.00–47.00]	49.00 [42.00–58.00]	<0.0001

Sex, *n* (%)			0.0387
Male	31 (36.9)	36 (53.7)	
Female	53 (63.1)	31 (46.3)	

MIRS, *n* (%)			0.0032
1	8 (9.5)	1 (1.5)	
2	26 (31)	10 (14.9)	
3	29 (34.5)	21 (31.3)	
4	19 (22.6)	33 (49.3)	
5	2 (2.4)	2 (3)	

Disease duration	11.00 [6.00–18.00]	14.00 [8.50–22.00]	0.0367
BMI	23.86 [21.30–25.85]	25.83 [23.39–30.00]	0.0017

CTG			
E1	17	8	
E2	55	56	
E3	11	3	
E4	1	0	

Sitters	2 (2.4)	2 (3)	0.8183
PM/ICD, *n* (%)	3 (3.6)	10 (14.9)	0.0184

All values are presented as median and interquartile range, except where otherwise indicated. ^*∗*^,^§^,° significance is indicated after Bonferroni correction. CTG expansion size was classified according to the scale of Tsilfidis and colleagues (Tsilfidis C, Mackenzie AE, Mettler G, et al. Correlation between CTG trinucleotide repeat length and frequency of severe congenital myotonic dystrophy. Nat Genet 1992; 1 : 192–5): E0, 38–79; E1, 80–499; E2, 500–999; E3, 1000–1499; E4, >1500. NIV, noninvasive ventilation; MIRS, Muscular Impairment Rating Scale; BMI, body mass index; PM, pacemaker; ICD, implantable cardiac defibrillator.

**Table 4:** Detailed respiratory features of the 2 cohorts according to NIV indication.

	*n*	Cohort 1 (*n* = 84)	*n*	Cohort 2 (*n* = 67)	*p* value
		No NIV indication		NIV indication at baseline	
		Median [IQR]		Median [IQR]	
Nocturnal oximetry					
Mean SpO2	65	96.00 [94.40–97.30]	63	92.40 [90.30–94.10]	<0.0001
Min SpO2	61	88.00 [84.00–92.00]	61	80.00 [71.00–84.00]	<0.0001
ODI	58	1.40 [0.20–3.80]	63	14.80 [6.30–21.70]	<0.0001

Morning ABG					
pH	59	7.47 [7.44–7.49]	62	7.43 [7.42–7.46]	<0.0001
pCO2	59	39.50 [35.60–43.80]	63	45.00 [42.00–48.30]	<0.0001
pO2	59	89.40 [78.60–101.60]	63	77.20 [67.40–85.20]	<0.0001
HCO3-	59	29.30 [26.70–31.60]	62	31.35 [28.60–33.00]	0.0116

Spirometry					
FVC % sitting	84	89.00 [74.50–99.50]	67	72.00 [59.00–88.00]	<0.0001
FVC % supine	78	83.80 [72.00–97.00]	53	68.00 [49.00–82.00]	<0.0001
FEV1%	70	81.00 [71.00–96.00]	59	72.00 [55.00–83.00]	0.0033
FEV1/FVC	70	0.80 [0.76–0.84]	59	0.81 [0.75–0.86]	0.4156
∆ FVC	78	4.00 [0.00–8.00]	53	8.00 [4.00–14.00]	0.0001
PCEF	84	343.50 [289.50–417.00]	65	303.80 [256.00–365.00]	0.0145
MIP	40	61.50 [44.00–89.50]	18	43.00 [28.00–60.00]	0.0253
MEP	40	79.00 [53.00–103.00]	18	58.00 [36.00–75.00]	0.1047

6MWT					
Meters	60	458.50 [390.00–528.00]	43	315.00 [240.00–375.00]	<0.0001
Mean O2 saturation	47	97.00 [95.00–98.00]	27	93.00 [88.00–95.00]	<0.0001
Pretest dyspnea	51	0.00 [0.00–0.00]	27	0.00 [0.00–2.00]	0.2901
Post-test dyspnea	51	2.00 [1.00–3.00]	27	3.00 [2.00–5.00]	0.1515

Secretion management					
Air stacking	9	(10.7)^*∗*^	6	(9)^*∗*^	0.7897
Cough assist	2	(2.4)^*∗*^	6	(9)^*∗*^	0.1395

IQR, interquartile range; SpO2, oxygen saturation; ODI, Oxygen Desaturation Index; pH, potential of hydrogen; pCO2, partial pressure of carbon dioxide; pO2, partial pressure of oxygen; HCO3-, bicarbonate; FVC, forced vital capacity; FEV1, forced expiratory volume in 1 second; PCEF, peak cough expiratory flow; MIP, maximal inspiratory pressure; MEP, maximal expiratory pressure; 6MWT, six-minute walk test; NIV, noninvasive ventilation. Pretest dyspnea and post-test dyspnea were calculated using the modified dyspnea Borg scale. This scale asks the patient to rate the difficulty of breathing from 0 (no difficulty) to 10 (maximal difficulty) at the very beginning of the 6MWT and immediately after. ^*∗*^percentage.

**Table 5:** Changes per year of respiratory features from the cohort available for follow-up.

	*n*	Overall cohort (*n* = 116)
		Median [IQR]
Follow-up period (months)	116	44.90 [26.32–59.20]

Nocturnal oximetry		
Mean SpO2	91	−0.02 [−0.50–0.52]
Min SpO2	83	0.00 [−1.39–1.73]
ODI	82	−0.40 [−2.78–0.28]

Morning arterial blood gas analysis		
pH	66	0.01 [0.00–0.02]
pCO2	68	−0.59 [−1.42–0.86]
pO2	66	−0.13 [−2.94–2.86]
HCO3-	65	−0.05 [−0.75–1.05]

Spirometry		
FVC % sitting	115	−0.99 [−3.98–1.34]
FVC % supine	52	−0.72 [−2.58–2.09]
FEV1%	96	−0.54 [−3.21–2.88]
FEV1/FVC	96	0.02 [−0.01–0.04]
∆ FVC	52	−0.40 [−2.78–1.86]
PCEF	109	6.77 [−4.22–22.08]
MIP	20	1.51 [−4.19–9.83]
MEP	20	1.22 [−9.37–18.90]

6MWT		
Meters	65	4.04 [−9.36–18.90]
Mean O2 saturation	45	−0.22 [−1.09–0.54]
Pretest dyspnea	48	0.00 [0.00–0.12]
Post-test dyspnea	45	0.00 [0.00–1.18]

IQR, interquartile range; SpO2, oxygen saturation; ODI, Oxygen Desaturation Index; pH, potential of hydrogen; pCO2, partial pressure of carbon dioxide; pO2, partial pressure of oxygen; HCO3-, bicarbonate; FVC, forced vital capacity; FEV1, forced expiratory volume in 1 second; PCEF, peak cough expiratory flow; MIP, maximal inspiratory pressure; MEP, maximal expiratory pressure; 6MWT, six-minute walk test; NIV, noninvasive ventilation. Pretest dyspnea and post-test dyspnea were calculated using the modified dyspnea Borg scale. This scale asks the patient to rate the difficulty of breathing from 0 (no difficulty) to 10 (maximal difficulty) at the very beginning of the 6MWT and immediately after.

**Table 6:** Changes per year of the respiratory parameters in the 2 cohorts according to NIV indication.

	*n*	Cohort 1 (*n* = 59)	*n*	Cohort 2 (*n* = 57)	*p* value
		No NIV indication		NIV indication at baseline	
		Median [IQR]		Median [IQR]	
Follow-up period (months)	59	38.10 [24.13–55.60]	57	46.17 [31.17–60.83]	0.0758

Nocturnal oximetry					
Mean SpO2	39	−0.34 [−0.61–0.04]	52	0.24 [−0.33–1.07]	0.0016
Min SpO2	35	0.27 [−0.74–1.30]	48	0.00 [−2.19–2.47]	0.9742
ODI	33	−0.02 [−0.53–0.71]	49	−2.13 [−4.07–−0.08]	0.0008

Morning ABG					
pH	28	0.00 [−0.01–0.02]	38	0.01 [0.00–0.02]	0.1966
pCO2	30	−0.09 [−1.34–1.72]	38	−0.97 [−1.91–0.00]	0.0342
pO2	29	−1.46 [−5.11–3.41]	37	0.26 [−1.96–2.59]	0.5523
HCO3-	28	0.31 [−1.13–1.49]	37	−0.16 [−0.68–0.59]	0.5035

Spirometry					
FVC % sitting	58	−1.17 [−3.98–1.43]	57	−0.97 [−3.95–1.19]	0.9354
FVC % supine	42	−0.79 [−2.72–1.97]	10	−0.58 [−2.37–5.56]	0.5081
FEV1%	46	−0.91 [−2.68–2.96]	50	−0.33 [−3.87–2.81]	0.9124
FEV1/FVC	46	0.03 [0.01–0.06]	50	0.00 [−0.01–0.02]	<0.0001
∆ FVC	42	−0.40 [−2.67–2.23]	10	−0.66 [−5.91–0.20]	0.4505
PCEF	58	10.88 [2.99–30.61]	51	1.38 [−17.49–16.12]	0.0048
MIP	14	2.89 [−4.85–17.37]	6	1.51 [−3.53–1.84]	0.5362
MEP	14	−0.01 [−10.10–35.66]	6	2.02 [−1.57–12.45]	0.9671

6MWT					
Meters	37	5.09 [−9.61–18.90]	28	2.89 [−8.18–22.48]	0.9525
Mean O2 saturation	28	−0.34 [−1.07–0.08]	17	0.26 [−1.12–0.77]	0.2101
Pretest dyspnea	30	0.00 [0.00–0.00]	18	0.00 [−0.52–0.24]	0.6302
Post-test dyspnea	28	0.00 [−0.12–1.19]	17	0.29 [0.00–1.18]	0.7490

IQR, interquartile range; SpO2, oxygen saturation; ODI, Oxygen Desaturation Index; pH, Potential of Hydrogen; pCO2, partial pressure of carbon dioxide; pO2, partial pressure of oxygen; HCO3-, bicarbonate; FVC, forced vital capacity; FEV1, forced expiratory volume in 1 second; PCEF, peak cough expiratory flow; MIP, maximal inspiratory pressure; MEP, maximal expiratory pressure; 6MWT, six-minute walk test; NIV, noninvasive ventilation. Pretest dyspnea and post-test dyspnea were calculated using the modified dyspnea Borg scale. This scale asks the patient to rate the difficulty of breathing from 0 (no difficulty) to 10 (maximal difficulty) at the very beginning of the 6MWT and immediately after.

**Table 7:** Changes per year in the patients' respiratory parameters based on their NIV adherence.

		NIV nonadherent (*n* = 40)		NIV adherent (*n* = 17)	*p* value
Follow-up period (months)	40	48.20 [31.50–61.35]	17	46.17 [31.17–59.73]	0.6691

Nocturnal oximetry					
Mean SpO2	36	0.00 [−0.47–0.71]	16	0.68 [0.07–2.90]	0.0198
Min SpO2	35	0.00 [−2.61–0.99]	13	3.12 [1.34–4.87]	0.0093
ODI	34	−0.41 [−3.04–0.14]	15	−3.66 [−6.88–−2.64]	0.0033

Morning ABG					
pH	27	0.01 [0.00–0.02]	11	0.01 [0.00–0.02]	0.9743
pCO2	27	−1.08 [−1.81–0.00]	11	−0.55 [−2.67–0.04]	0.8469
pO2	27	−0.82 [−2.94–1.80]	10	2.72 [0.26–6.12]	0.0175
HCO3-	27	−0.05 [−0.67–1.05]	10	−0.30 [−1.04–0.24]	0.1879

Spirometry					
FVC % sitting	40	−1.67 [−4.35 to −0.13]	17	0.92 [−0.85–2.29]	0.0273
FVC % supine	9	−0.66 [−2.37–1.98]	0		
FEV1%	36	−0.47 [−4.00–2.44]	14	0.36 [−1.72–2.81]	0.4055
FEV1/FVC	36	0.00 [−0.01–0.02]	14	0.00 [−0.02–0.02]	0.6198
∆ FVC	9	0.00 [−2.84–0.20]	0		
PCEF	34	4.12 [−12.43–16.17]	17	−2.66 [−18.96–15.39]	0.5690
MIP	5	1.35 [−3.53–1.66]	0		
MEP	5	−0.71 [−1.57–4.74]	0		

6MWT					
Meters	19	5.36 [−7.86–18.07]	9	−2.42 [−42.63–28.80]	0.5225
Mean O2 saturation	11	0.50 [−1.12–2.19]	6	0.13 [−1.12–0.60]	0.6511
Pretest dyspnea	12	0.00 [−0.28–0.26]	6	0.0 [−0.52–0.00]	0.7688
Post-test dyspnea	11	0.29 [0.00–1.51]	6	0.26 [0.00–0.52]	0.6470

IQR, interquartile range; SpO2, oxygen saturation; ODI, Oxygen Desaturation Index; pH, potential of hydrogen; pCO2, partial pressure of carbon dioxide; pO2, partial pressure of oxygen; HCO3-, bicarbonate; FVC, forced vital capacity; FEV1, forced expiratory volume in 1 second; PCEF, peak cough expiratory flow; MIP, maximal inspiratory pressure; MEP, maximal expiratory pressure; 6MWT, six-minute walk test; NIV, noninvasive ventilation. Pretest dyspnea and post-test dyspnea were calculated using the modified dyspnea Borg scale. This scale asks the patient to rate the difficulty of breathing from 0 (no difficulty) to 10 (maximal difficulty) at the very beginning of the 6MWT and immediately after.

## Data Availability

All data generated or analyzed during this study are included in this published article (and its supplementary information files).

## Supplementary Materials

Supplementary Materials e-Table 1: Details of respiratory function for each patient at baseline and over time. e-Table 2. Correlations between respiratory parameters and demographic and clinical features.

## References

[1] Harper P. S. (2001). Myotonic dystrophy. *Major Problems in Neurology*.

[2] de Die-Smulders C., Höweler C. J., Thijs C. (1998). Age and causes of death in adult-onset myotonic dystrophy. *Brain*.

[3] Mathieu J., Allard P., Potvin L., Prévost C., Bégin P. (1999). A 10-year study of mortality in a cohort of patients with myotonic dystrophy. *Neurology*.

[4] Sansone V. A., Gagnon C. (2015). 207th ENMC Workshop on chronic respiratory insufficiency in myotonic dystrophies: management and implications for research, 27-29 June 2014, Naarden, The Netherlands. *Neuromuscular Disorders*.

[5] Thil C., Agrinier N., Chenuel B., Poussel M. (2017). Longitudinal course of lung function in myotonic dystrophy type 1. *Muscle & Nerve*.

[6] Hawkins A. M., Hawkins C. L., Abdul Razak K., Khoo T. K., Tran K., Jackson R. V. (2019). Respiratory dysfunction in myotonic dystrophy type 1: a systematic review. *Neuromuscular Disorders*.

[7] Seijger C. G. W., Drost G., Posma J. M., van Engelen B. G. M., Heijdra Y. F. (2016). Overweight is an independent risk factor for reduced lung volumes in myotonic dystrophy type 1. *PLoS One*.

[8] Boussaïd G., Prigent H., Laforet P. (2018). Effect and impact of mechanical ventilation in myotonic dystrophy type 1: a prospective cohort study. *Thorax*.

[9] Boussaïd G., Wahbi K., Laforet P. (2018). Genotype and other determinants of respiratory function in myotonic dystrophy type 1. *Neuromuscular Disorders*.

[10] Monteiro R., Bento J., Gonçalves M. R., Pinto T., Winck J. C. (2013). Genetics correlates with lung function and nocturnal ventilation in myotonic dystrophy. *Sleep and Breathing*.

[11] Rossi S., Della Marca G., Ricci M. (2019). Prevalence and predictor factors of respiratory impairment in a large cohort of patients with myotonic dystrophy type 1 (DM1): a retrospective, cross sectional study. *Journal of the Neurological Sciences*.

[12] Solbakken G., Bjørnarå B., Kirkhus E. (2019). MRI of trunk muscles and motor and respiratory function in patients with myotonic dystrophy type 1. *BMC Neurology*.

[13] Graham B. L., Steenbruggen I., Miller M. R. (2019). Standardization of spirometry 2019 update. An official American thoracic society and European respiratory society technical statement. *American Journal of Respiratory and Critical Care Medicine*.

[14] Babačić H., Goldina O., Stahl K. (2018). How to interpret abnormal findings of spirometry and manometry in myotonic dystrophies?. *Journal of Neuromuscular Diseases*.

[15] Chatwin M., Ross E., Hart N., Nickol A. H., Polkey M. I., Simonds A. K. (2003). Cough augmentation with mechanical insufflation/exsufflation in patients with neuromuscular weakness. *European Respiratory Journal*.

[16] Annane D., Orlikowski D., Chevret S., Chevrolet J. C., Raphaël J. C. (2007). Nocturnal mechanical ventilation for chronic hypoventilation in patients with neuromuscular and chest wall disorders. *The Cochrane Database of Systematic Reviews*.

[17] Poussel M., Thil C., Kaminsky P. (2015). Lack of correlation between the ventilatory response to CO_2_ and lung function impairment in myotonic dystrophy patients: evidence for a dysregulation at central level. *Neuromuscular Disorders*.

[18] Boentert M., Cao M., Mass D. (2020). Consensus-based care recommendations for pulmonologists treating adults with myotonic dystrophy type 1. *Respiration*.

[19] Boussaïd G., Lofaso F., Santos D. B. (2016). Factors influencing compliance with non-invasive ventilation at long-term in patients with myotonic dystrophy type 1: a prospective cohort. *Neuromuscular Disorders*.

[20] Fiorentino G., Esquinas A. M. (2018). Non invasive mechanical ventilation in myotonic dystrophy type 1? Hypoventilation versus quality of life perspective. *Neuromuscular Disorders*.

[21] Spiesshoefer J., Runte M., Heidbreder A. (2019). Sleep-disordered breathing and effects of non-invasive ventilation on objective sleep and nocturnal respiration in patients with myotonic dystrophy type I. *Neuromuscular Disorders*.

[22] Miller M. R., Hankinson J., Brusasco V. (2005). Standardisation of spirometry. *European Respiratory Journal*.

[23] Laveneziana P., Albuquerque A., Aliverti A. (2019). ERS statement on respiratory muscle testing at rest and during exercise. *European Respiratory Journal*.

[24] Kapur V. K., Auckley D. H., Chowdhuri S. (2017). Clinical practice guideline for diagnostic testing for adult obstructive sleep apnea: an American academy of sleep medicine clinical practice guideline. *Journal of Clinical Sleep Medicine*.

[25] Vignatelli L., Plazzi G., Barbato A. (2003). Italian version of the Epworth sleepiness scale: external validity. *Neurological Sciences*.

[26] Mathieu J., Boivin H., Meunier D., Gaudreault M., Bégin P. (2001). Assessment of a disease-specific muscular impairment rating scale in myotonic dystrophy. *Neurology*.

[27] Caricati L., Foà C., Fruggeri L., Tonarelli A. (2015). COPE-NVI-25: validazione italiana della versione ridotta della coping orientation to the Problems Experienced (COPE-NVI). *Psicologia della Salute*.

[28] Baldanzi S., Bevilacqua F., Lorio R. (2016). Disease awareness in myotonic dystrophy type 1: an observational cross-sectional study. *Orphanet Journal of Rare Diseases*.

[29] LaDonna K. A., Ghavanini A. A., Venance S. L. (2015). Truths and misinformation: a qualitative exploration of myotonic dystrophy. *The Canadian Journal of Neurological Sciences*.

[30] Bianchi M. L. E., Losurdo A., Di Blasi C. (2014). Prevalence and clinical correlates of sleep disordered breathing in myotonic dystrophy types 1 and 2. *Sleep and Breathing*.

[31] Laberge L., Begin P., Montplaisir J., Mathieu J. (2004). Sleep complaints in patients with myotonic dystrophy. *Journal of Sleep Research*.

[32] O’Donoghue F. J., Borel J.-C., Deauvilliers Y., Levy P., Tamisier R., Pépin J. L. (2017). Effects of 1-month withdrawal of ventilatory support in hypercapnic myotonic dystrophy type 1. *Respirology*.

[33] Mazzoli M., Ariatti A., Garuti G. C. (2020). Predictors of prognosis in type 1 myotonic dystrophy (DM1): longitudinal 18-years experience from a single center. *Acta Myologica: Myopathies and Cardiomyopathies: Official Journal of the Mediterranean Society of Myology*.

