# Supplementary material for: Assessment of Respiratory Function and Need for Noninvasive Ventilation in a Cohort of Patients with Myotonic Dystrophy Type 1 Followed at One Single Expert Center
Source: Can Respir J. 2022 Jun 18;2022:2321909. doi: 10.1155/2022/2321909 (PMC9233572; doi:10.1155/2022/2321909)
Supplement: Supplementary Materials — e-Table 1: Details of respiratory function for each patient at baseline and over time. e-Table 2. Correlations between respiratory parameters and demographic and clinical features. [file 2321909.f1.zip › 2321909.f1/eTable 2.docx]

| **eTable 2. Correlations between respiratory parameters and demographic and clinical features** | | | | | | |
| --- | --- | --- | --- | --- | --- | --- |
|  | **Age** | | **Disease Duration** | | **BMI** | |
|  | ***rho*** | **p-value** | ***rho*** | **p-value** | ***rho*** | **p-value** |
| **Nocturnal oximetry** |  |  |  |  |  |  |
| Mean SpO2 | -0.45 | <.0001 | -0.23 | 0.0117 | -0.41 | <.0001 |
| Min SpO2 | -0.39 | <.0001 | -0.26 | 0.0068 | -0.36 | <.0001 |
| ODI | 0.39 | <.0001 | 0.21 | 0.0297 | 0.32 | 0.0003 |
| **Morning ABG** |  |  |  |  |  |  |
| pH | 0.04 | 0.6440 | -0.15 | 0.1167 | -0.08 | 0.4060 |
| pCO2 | 0.14 | 0.1379 | 0.01 | 0.9407 | -0.04 | 0.6948 |
| pO2 | -0.32 | 0.0003 | -0.10 | 0.3137 | -0.29 | 0.0013 |
| HCO3- | 0.13 | 0.1439 | -0.12 | 0.2302 | -0.10 | 0.2792 |
| **Spirometry** | | | | | |  |
| FVC % sitting | -0.17 | 0.0328 | -0.27 | 0.0182 | -0.24 | 0.0027 |
| FVC % supine | -0.20 | 0.0206 | -0.22 | 0.0182 | -0.29 | 0.0009 |
| FEV1 % | -0.06 | 0.5002 | -0.15 | 0.1116 | -0.14 | 0.1164 |
| FEV1/FVC | -0.01 | 0.9767 | 0.11 | 0.2413 | 0.08 | 0.3816 |
| ∆ FVC % | 0.14 | 0.1207 | 0.06 | 0.5486 | 0.16 | 0.0662 |
| PCEF | -0.19 | 0.0191 | -0.13 | 0.1264 | 0.06 | 0.4765 |
| MIP | -0.17 | 0.1962 | -0.17 | 0.2186 | -0.09 | 0.5076 |
| MEP | -0.09 | 0.5125 | -0.13 | 0.3511 | 0.06 | 0.6502 |
| **6MWT** | | | | | |  |
| Meters | -0.52 | <.0001 | -0.20 | 0.0502 | -0.34 | 0.0005 |
| Mean O2 saturation | -0.35 | 0.0019 | 0.02 | 0.8555 | -0.38 | 0.0009 |
| Pre-test dyspnea | 0.12 | 0.3021 | -0.17 | 0.1416 | 0.06 | 0.6055 |
| Post-test dyspnea | 0.12 | 0.2806 | 0.03 | 0.8205 | 0.15 | 0.1834 |

*Abbreviations: IQR, Interquartile range; SpO2, oxygen saturation; ODI, Oxygen Desaturation Index; pH, Potential of Hydrogen; pCO2, partial pressure of carbon dioxide; pO2, partial pressure of oxygen; HCO3-, Bicarbonate; FVC, Forced Vital Capacity; FEV1, Forced Expiratory Volume in 1 second; PCEF, Peak Cough Expiratory Flow; MIP, Maximal Inspiratory Pressure; MEP, Maximal Expiratory Pressure; 6MWT, six-minute walking test; NIV, non-invasive ventilation.*

*Pre- and post-test dyspnea were calculated using the modified dyspnea Borg scale. This scale asks the patient to rate the difficulty of breathing from 0 (no difficulty) to 10 (maximal difficulty) at the very beginning of the 6MWT and immediately after.*
